# TCAD and Garfield++ Optimisation of Single-Electrode Silicon Electron Multiplier Sensors

**DOI:** 10.3390/s26175424

**Published:** 2026-08-27

**Authors:** Federico De Benedetti, Victor Coco, Paula Collins, Abraham Gallas Torreira, Edgar Lemos Cid, Alfonso Puicercus Gomez, Allan da Silva Jales, Heinrich Schindler

**Affiliations:** 1 CERN, 1217 Geneva, Switzerland; 2 Instituto Galego de Física de Altas Enerxías (IGFAE) and Universidade de Santiago de Compostela (USC), 15705 Santiago de Compostela, Spain; 3 Faculty of Science and Engineering, University of Groningen, 9747 AG Groningen, The Netherlands; 4 Universidade do Estado do Rio de Janeiro (UERJ), Rio de Janeiro 20550-900, Brazil

**Keywords:** ultra-fast silicon detectors, timing detectors, TCAD, Garfield++

## Abstract

The Silicon Electron Multiplier (SiEM) is a novel silicon sensor concept for minimum-ionising particle (MIP) detection in which internal gain is achieved through a composite electrode structure embedded in the silicon bulk. Unlike conventional gain sensors such as Low-Gain Avalanche Detectors (LGADs), the SiEM produces internal multiplication through electrostatic field shaping rather than through doped gain layers. This architecture is also finely segmented by design, with a pixel pitch of 10 μm, making it a promising candidate for small pitch radiation-hard tracking and timing applications. This work extends the initial conceptual studies by using a three-dimensional Technology Computer-Aided Design (TCAD) and Garfield++ simulation pipeline for simplified single-electrode SiEM geometries. The study evaluates how the multiplication-region geometry affects electrical response, effective charge collection, internal gain, intrinsic timing performance, and the fraction of the pixel area providing a full MIP response. The results show that enlarging the multiplication pillar can improve the effective active area while preserving timing performance comparable to the initial conceptual design.

## 1. Introduction

### 1.1. Motivations and Challenges

Future high-energy physics experiments, such as those at the High-Luminosity Large Hadron Collider (HL-LHC) and at proposed future hadron colliders, will operate under increasingly challenging radiation and pile-up conditions [1]. The innermost tracking detectors will therefore require sensors combining high radiation tolerance, fine spatial segmentation, and precise timing performance. Conventional silicon technologies involve intrinsic trade-offs between these requirements.

Thin planar sensors can withstand fluences approaching $10^{16}$ $1\;\mathrm{MeV}\;n_{\mathrm{eq}}\;{\mathrm{cm}}^{-2}$, but their limited signal amplitude constrains the achievable timing performance [2]. 3D silicon sensors reduce carrier drift distances and can achieve intrinsic time resolutions down to 20 ps to 30 ps, but at the cost of increased capacitance, reduced fill factor, and technological complexity at very small pixel pitches [3,4]. Low-Gain Avalanche Detectors (LGADs) provide excellent timing through controlled internal avalanche multiplication, with time resolutions around 20 ps to 30 ps [5], but their gain layer is affected by acceptor removal after irradiation, limiting their performance at high fluence [6]. These limitations motivate the investigation of alternative silicon sensor concepts capable of combining internal gain and radiation tolerance with sub-micron spatial resolution enabled by fine sensor segmentation.

### 1.2. The SiEM Working Principle

The Silicon Electron Multiplier (SiEM) is a silicon sensor concept designed to combine internal gain with ultra-fine pixel segmentation at 10 μm pitch.

**Figure 1:**
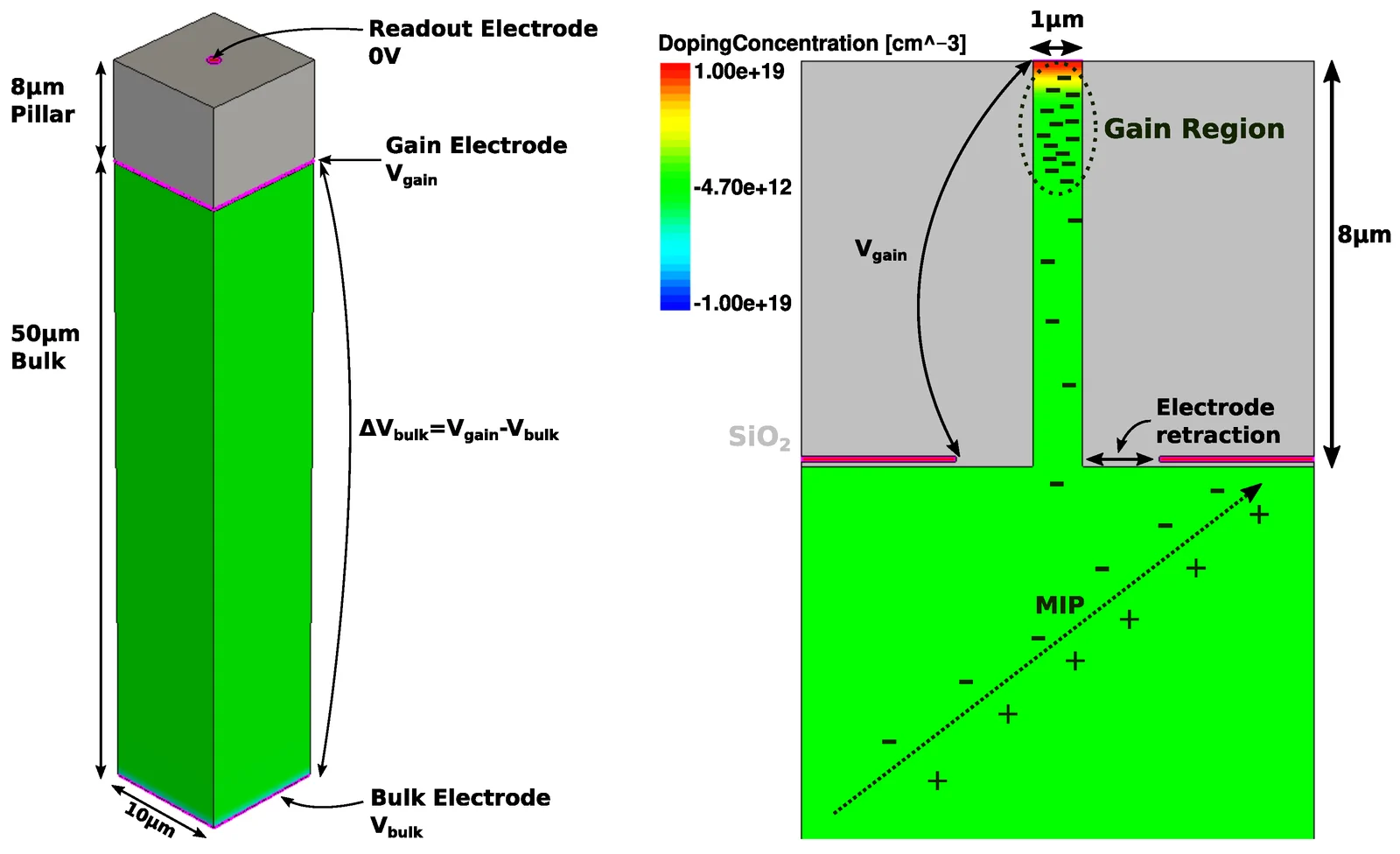
Conceptual layout of the single-electrode Silicon Electron Multiplier (SiEM) considered in this work. Left: Three-dimensional view of the simulated unit cell, showing the depleted silicon bulk, readout electrode, and embedded gain structure. Right: Cross-section of the pillar region, where the high electric field is localised and internal avalanche multiplication occurs.

The basic operating principle is illustrated in Figure 1. Here, a 50 μm thick depleted silicon bulk acts as the drift region, where charge carriers generated by a traversing minimum-ionising particle (MIP) drift towards a set of silicon pillars in which a high electric field is generated. This field is established by applying a voltage either between two sets of buried electrodes (double-electrode configuration) or between the buried electrode and the readout electrode (single-electrode configuration), made of aluminium.

In the single-electrode configuration considered here, the embedded gain electrode shapes a high electric field inside the pillar, producing internal avalanche multiplication via impact ionisation without relying on the doped gain layer used in LGADs. Charge multiplication is therefore controlled primarily by the device geometry and bias voltage, potentially reducing the sensitivity to radiation-induced dopant removal.

### 1.3. Scope of This Work

The first study introducing the SiEM concept established the proof of concept, reporting gains above 10 and an intrinsic timing performance of about 40 ps for 50 μm thick sensors [7]. In that work, single- and double-electrode configurations were explored over a broad design phase space using mainly Technology Computer-Aided Design (TCAD) simulations, providing a first assessment of the role of electrode layout and biasing scheme.

This work extends that study by combining 3D TCAD field maps with Garfield++ Monte Carlo transient simulations, which describe realistic MIP charge deposition, carrier transport, and signal formation over many particle impact positions across the pixel. This enables the evaluation of pixel efficiency and timing uniformity.

The study focuses on fabrication-aware SiEM geometries intended for future demonstrators to be developed with the Centro Nacional de Microelectrónica (CNM).

Following the initial conceptual study [7], several production paths were investigated for the fabrication of SiEM demonstrators [8,9,10]. In ref. [9], a process based on Deep Reactive Ion Etching (DRIE) was developed at CNM. The geometry, material and doping parameters used in this work derive from this process.

The initial CNM demonstrator design was based on the double-electrode SiEM architecture introduced in [7]. There, a 1 μm diameter pillar was found to provide strong field localisation and favourable multiplication behaviour. However, the CNM fabrication process, in particular the laser photolithography step, limits the minimum achievable pillar diameter to approximately 2 μm. For this reason, tapered pillars were introduced as a possible way to preserve a 1 μm multiplication region at the pillar bottom while keeping the top pillar diameter compatible with fabrication constraints.

In this work, the focus is shifted to the single-electrode architecture, which reduces the number of embedded conductive layers and associated fabrication steps with respect to the double-electrode layout. Since this configuration leads to a different electric-field distribution, it is necessary to verify whether preserving a 1 μm multiplication region is still beneficial. Enlarged cylindrical and tapered pillar geometries are therefore studied to assess their impact on gain, effective active area, and timing performance.

## 2. Investigated Geometries

The investigated geometries are defined within the design space motivated above. Each simulated cell consists of a 50 μm thick p-type silicon bulk, a top readout contact, and an embedded gain electrode surrounding an n^+^ multiplication pillar, with a square pitch of 10 μm. The bulk is doped to a concentration of $4.7\times 10^{12}{\mathrm{cm}}^{-3}$, and the n^+^ pillar to a phosphorus concentration of $1\times 10^{19}{\mathrm{cm}}^{-3}$, localised at the pillar tip with a Gaussian doping profile, consistent with the values used in [7]. The readout electrode is kept at ground potential, while the bulk and gain electrode are biased independently to ensure substrate depletion and formation of the high-field multiplication region.

The reference geometry is a cylindrical pillar with 1 μm diameter and 50 nm electrode retraction, corresponding to the geometry introduced in [7]. Cylindrical pillars with diameters from 2 μm to 4 μm are considered to study the effect of increasing the multiplication-region size. Tapered pillars are modelled as truncated cones with a fixed 1 μm bottom diameter and top diameters from 2 μm to 4 μm. This choice allows geometries preserving a 1 μm pillar base to be directly compared with cylindrical geometries of the same top diameter.

The gain-electrode retraction is defined as the lateral distance between the pillar top edge and the inner edge of the surrounding gain electrode. For the 1 μm reference geometry, the retraction is varied from 50 nm to 1.5 μm to study the effect of the gain-electrode position independently of the pillar diameter.

All geometries share a 10 μm square pitch, a 50 μm thick sensor, and an 8 μm pillar height. Although these parameters are expected to influence the detector response, they are kept fixed at the baseline values foreseen for the first CNM fabrication run. The study is therefore restricted to variations of the pillar diameter, pillar shape, and gain-electrode retraction.

Two representative configurations are discussed in detail in the following sections: the baseline 1 μm cylindrical pillar and an enlarged 4 μm cylindrical pillar with the same nominal electrode retraction. The full set of simulated geometries is summarised in Table 1.

## 3. Simulation Methods

The simulations are performed with Synopsys Sentaurus TCAD [11], version S-2023.12, and Garfield++ [12]. Device geometries are defined in Sentaurus Device Editor (SDE), while Sentaurus Device (SDEVICE) is used to compute the electrostatic solutions, I–V and C–V characteristics, and electric-field maps of the different layouts. A finer mesh is applied near abrupt doping transitions and within the multiplication pillar to accurately resolve the strong electric-field gradients and localised impact-ionisation processes. The resulting TCAD mesh and electric-field maps are then processed and converted into a Garfield++-compatible format for charge-transport and signal-formation simulations.

**Figure 2:**
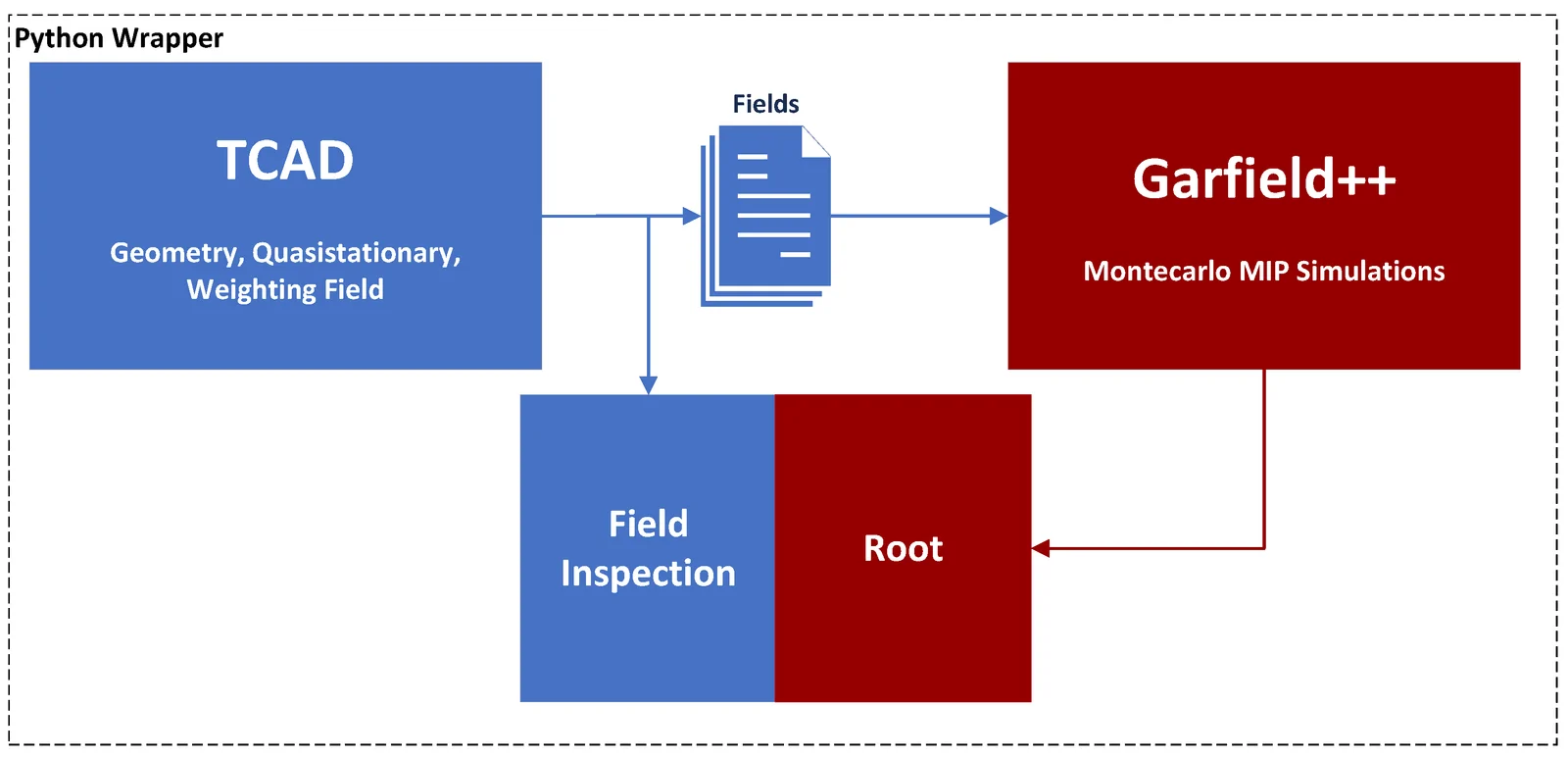
Simulation pipeline from TCAD geometry generation and quasistationary device simulation to field-map conversion and Garfield++ transient signal simulation.

A dedicated Python 3.9 wrapper was developed to generate the TCAD input files, launch the quasistationary and weighting field simulations in Sentaurus TCAD, and subsequently launch the Garfield++ simulations using the field maps exported by TCAD in .grd and .dat formats, natively supported by Garfield++. The complete simulation pipeline is shown in Figure 2.

### 3.1. Quasistationary Simulations

The electrical behaviour of each geometry is simulated in quasistationary mode with SDEVICE by solving the coupled Poisson and carrier-continuity equations along the applied bias ramp. The physical model set includes Fermi–Dirac statistics, Klaassen mobility with high-field saturation [13], Shockley–Read–Hall recombination with doping and temperature dependence [14], Hurkx field-enhanced recombination [15], Van Overstraeten–De Man impact ionisation [16], Slotboom band-gap narrowing [17], and an effective intrinsic carrier-density model. All simulations are performed at a fixed temperature of 300 K.

The biasing sequence is performed in two steps. First, the bulk contact is ramped to −50 V to fully deplete the 50 μm substrate, while the readout and gain electrodes are kept at ground. The gain scan is then performed by ramping the gain electrode while shifting the bulk potential to keep the bulk–gain voltage difference ($\Delta V_{\mathrm{bulk}}$) fixed at 50 V, with the readout electrode remaining grounded.

At each voltage step, the SDEVICE solution is used to extract the I–V and C–V characteristics. Two-dimensional electric-field maps are generated every 5 V to study the evolution of the multiplication region. The scan is stopped when the solver fails to converge or when the bulk current exceeds 10 nA, corresponding to the breakdown regime.

For selected bias points, corresponding to the highest stable gain voltage before breakdown, the weighting field is obtained by perturbing the readout electrode by 1 V and re-solving the electrostatic solution, following the extended Ramo–Shockley formalism for biased semiconductor detectors [18]. The nominal and weighting-field maps are then exported for Garfield++ for Monte Carlo simulations.

### 3.2. Transient Simulations

Signal formation is simulated in Garfield++ using TCAD electric-field and weighting-field maps rather than full 3D transient TCAD simulations. This choice is motivated both by the computational cost of scanning many particle impact positions and geometries in TCAD, and by the need for a Monte Carlo description of realistic MIP charge deposition and transport. In Garfield++, charge carriers are drifted in the imported TCAD fields, the induced current is computed from the weighting field, and impact ionisation is modelled using the Van Overstraeten–De Man parameterisation.

For each simulated event, a straight MIP trajectory is generated through the depleted bulk at normal incidence (ϕ=θ=0) and at a randomised (x,y) position within the pixel cell. Primary ionisation is simulated with the HEED package, which implements the Photo Absorption Ionisation (PAI) model to generate realistic ionisation clusters and energy-loss fluctuations along the particle trajectory.

### 3.3. Simulation Observables

The performance of the investigated layouts is evaluated through a set of observables extracted from both TCAD and Garfield++ simulations. The TCAD observables include the I–V and C–V characteristics, the electric-field distribution, the impact-ionisation map, and the electron-velocity streamlines. The Garfield++ simulations are then used to extract the collected charge, the effective charge collection efficiency, the area efficiency, and the centroid-time response to MIP signals.

For each simulated track, the total deposited charge is defined as

(1) $$Q_{\mathrm{deposited}}=q\sum_{i}N_{eh,i},$$

where $N_{eh,i}$ is the number of electron–hole pairs generated in the *i*-th ionisation cluster. Electrons and holes are then drifted and the induced current on the readout electrode is integrated to obtain the collected charge $Q_{\mathrm{collected}}$. The effective pixel charge collection efficiency is defined as the ratio of collected to deposited charge as

(2) $$CCE_{\mathrm{eff}}=\frac{Q_{\mathrm{collected}}}{Q_{\mathrm{deposited}}},$$

and includes both transport efficiency and internal multiplication. Values of $CCE_{\mathrm{eff}}>1$ indicate regions of the pixel where charge multiplication is obtained.

The area efficiency is defined as the fraction of the pixel area for which $CCE_{\mathrm{eff}}\geq 1$,

(3) $$\eta_{\mathrm{area}}=\frac{A_{CCE_{\mathrm{eff}}\geq 1}}{A_{\mathrm{tot}}}.$$

Timing performance is evaluated using the current-weighted time centroid of the total induced current signal. The fluctuation of the signal centroid time is a key contribution to the intrinsic time resolution of silicon sensors [19], and is therefore used here as a simulation-level observable to compare the intrinsic timing response of different SiEM geometries independently of a specific front-end implementation. For each simulated track, the centroid time is defined as

(4) $$T_{\mathrm{centroid}}=\frac{\sum_{j=1}^{n}I_{j}\;T_{j}}{\sum_{j=1}^{n}I_{j}}.$$

where $I_{j}$ is the induced current at the time sample $T_{j}$. The distribution of $T_{\mathrm{centroid}}$ over all simulated tracks is then used to extract the intrinsic timing spread. Centroid-time maps are built over the full pixel area by considering only tracks with collected charge above the 100 $e^{-}$ threshold, which rejects signals with too few collected carriers to provide a meaningful centroid estimate. These maps are used to assess the spatial uniformity of the timing response across the pixel and to identify delayed peripheral regions, which limit the overall timing performance.

The timing resolution is quantified as the standard deviation of the centroid-time distribution both over the full pixel area, $\sigma_{t}$, and within a Region of Interest (ROI) corresponding to the physical pillar region, $\sigma_{t,\mathrm{ROI}}$. The comparison between these two quantities separates the global timing uniformity of the pixel from the local timing contribution of the multiplication structure.

Each geometry is simulated with an average track density of 1000 $\mathrm{track}{\mu m}^{-2}$.

## 4. Simulation Results

### 4.1. Electrical Response

**Figure 3:**
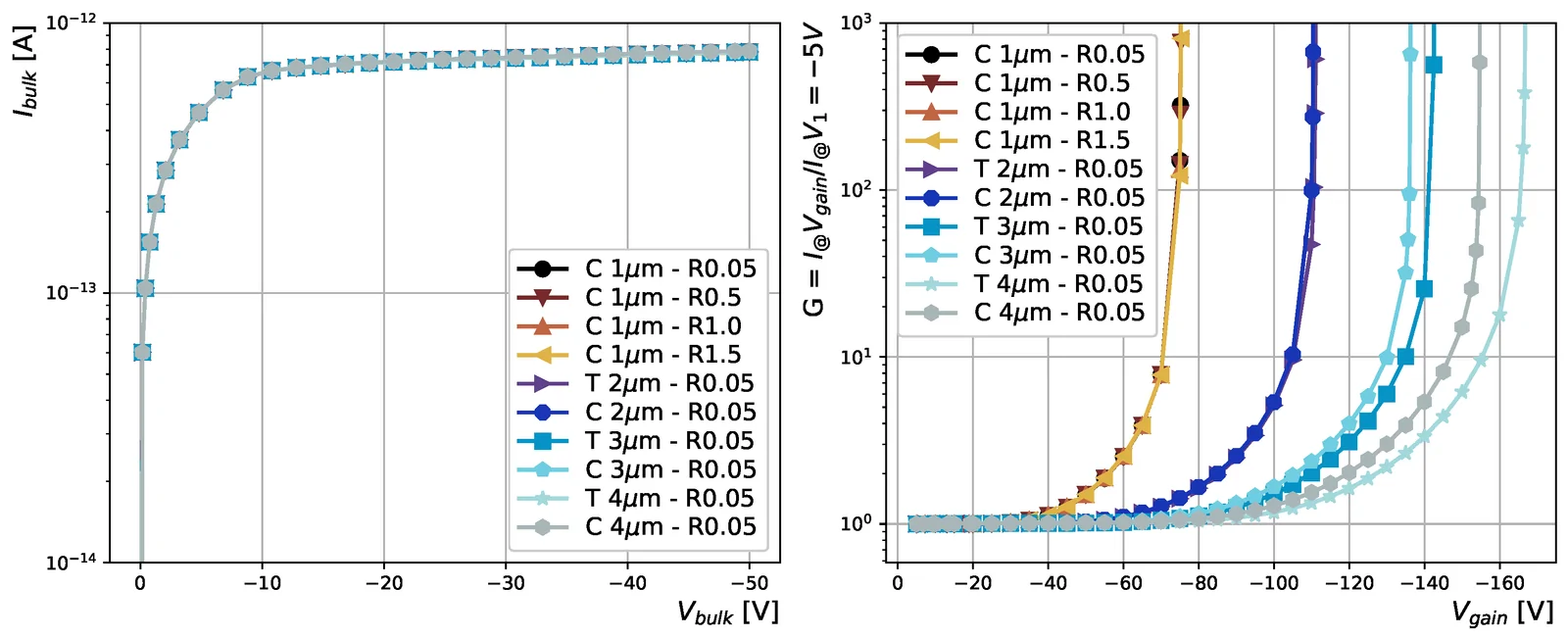
Simulated I–V characteristics of the investigated single-electrode SiEM geometries. (**Left**): Bulk-bias scan, showing the bulk leakage current as a function of the bulk voltage. All curves overlap in this plot, since the bulk depletion behaviour is independent of the multiplication-region geometry. (**Right**): Gain-bias scan, showing the normalised bulk leakage current as a function of the gain voltage. The curves for all C 1 μm geometries overlap, indicating that the gain onset and breakdown behaviour do not depend on the gain-electrode retraction. The comparison highlights the dependence of leakage current and gain onset on pillar width, tapering, and electrode retraction.

The simulated I–V characteristics for all investigated geometries are shown in Figure 3, where the bulk-bias and gain-bias scans are reported separately. The bulk-bias scan shows the bulk current as a function of the substrate voltage and is used to evaluate the depletion behaviour and leakage-current evolution. The gain-bias scan is normalised to the current value at the start of the gain ramp, allowing the relative current increase associated with multiplication to be isolated from the baseline leakage current.

The bulk-bias scan is similar for all structures, independently of the multiplication-region geometry, indicating comparable depletion behaviour. In the gain-bias scan, the bulk leakage current increases as the gain-electrode potential is raised, reflecting the onset and development of multiplication within the pillar, until breakdown is reached.

The smallest cylindrical geometries (C 1 μm) exhibit the earliest multiplication onset and approach avalanche breakdown at the lowest gain voltage. This is independent of the electrode retraction, indicating that the local field concentration is primarily determined by the pillar geometry rather than by the exact gain-electrode radial position. Increasing the pillar width shifts the multiplication onset and breakdown towards higher gain voltages, consistent with a broader and less localised high-field region.

**Figure 4:**
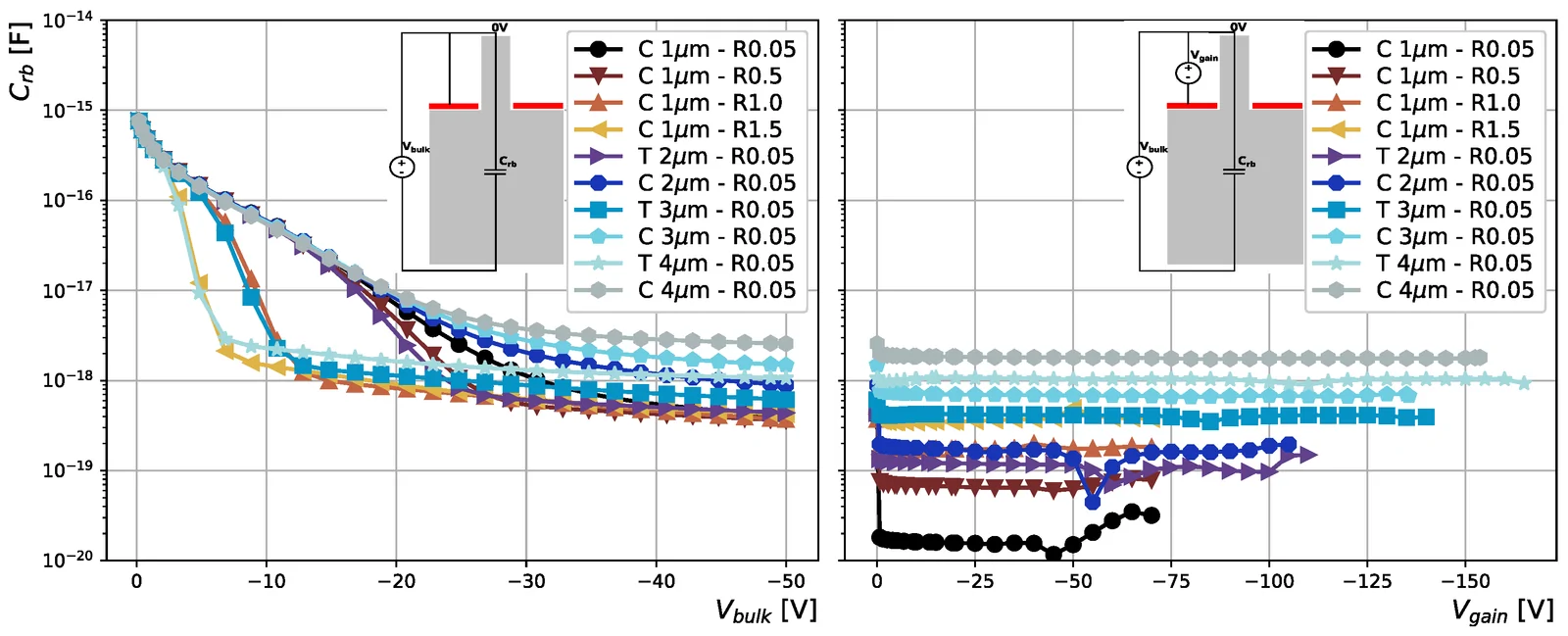
Simulated readout-to-bulk capacitance ($C_{rb}$) of the investigated single-electrode SiEM geometries, for the same set of geometries in both plots. The circuit schematics indicate that the capacitance is always evaluated between the readout and bulk electrodes, with $V_{\mathrm{gain}}=0$ in the left plot and $V_{\mathrm{gain}}$ actively biased in the right plot. (**Left**): $C_{rb}$ as a function of $V_{\mathrm{bulk}}$ during the bulk-bias ramp. (**Right**): $C_{rb}$ as a function of $V_{\mathrm{gain}}$ during the gain-electrode ramp. The capacitance exhibits a step when the gain electrode transitions from a grounded state to $V_{\mathrm{gain}}<0\;V$. The comparison highlights the dependence on electrode retraction, pillar width, and pillar shape.

The simulated readout-to-bulk capacitance is shown in Figure 4, where the bulk- and gain-bias scans are shown in separate plots. The capacitance exhibits a step when the gain electrode transitions from a grounded state to $V_{\mathrm{gain}}<0\;V$. As soon as the gain electrode is switched on, an electrostatic field is established between the gain electrode and the readout electrode, screening the pillar region and reducing the total capacitance. A moderate capacitance increase is observed as the electrode retraction increases. This behaviour can be attributed to the reduced electrostatic shielding provided by the gain electrode, which enhances the coupling between the readout electrode and the bulk. Comparing geometries with pillar diameters larger than 1 μm to the cylindrical reference geometry, the capacitance increases with pillar size because the larger readout-electrode area strengthens the electrostatic coupling to the bulk. This trend is observed for both cylindrical and tapered layouts, indicating that the top pillar diameter has a larger impact than the exact pillar shape.

Owing to the very small pixel pitch, the capacitance remains in the attofarad range for all investigated geometries. Such a low value is advantageous for front-end electronics, as it reduces the electronic-noise contribution and can therefore improve the achievable timing resolution by lowering the jitter term associated with the detector–electronics system [2,20].

### 4.2. Field Configuration and Multiplication Region

**Figure 5:**
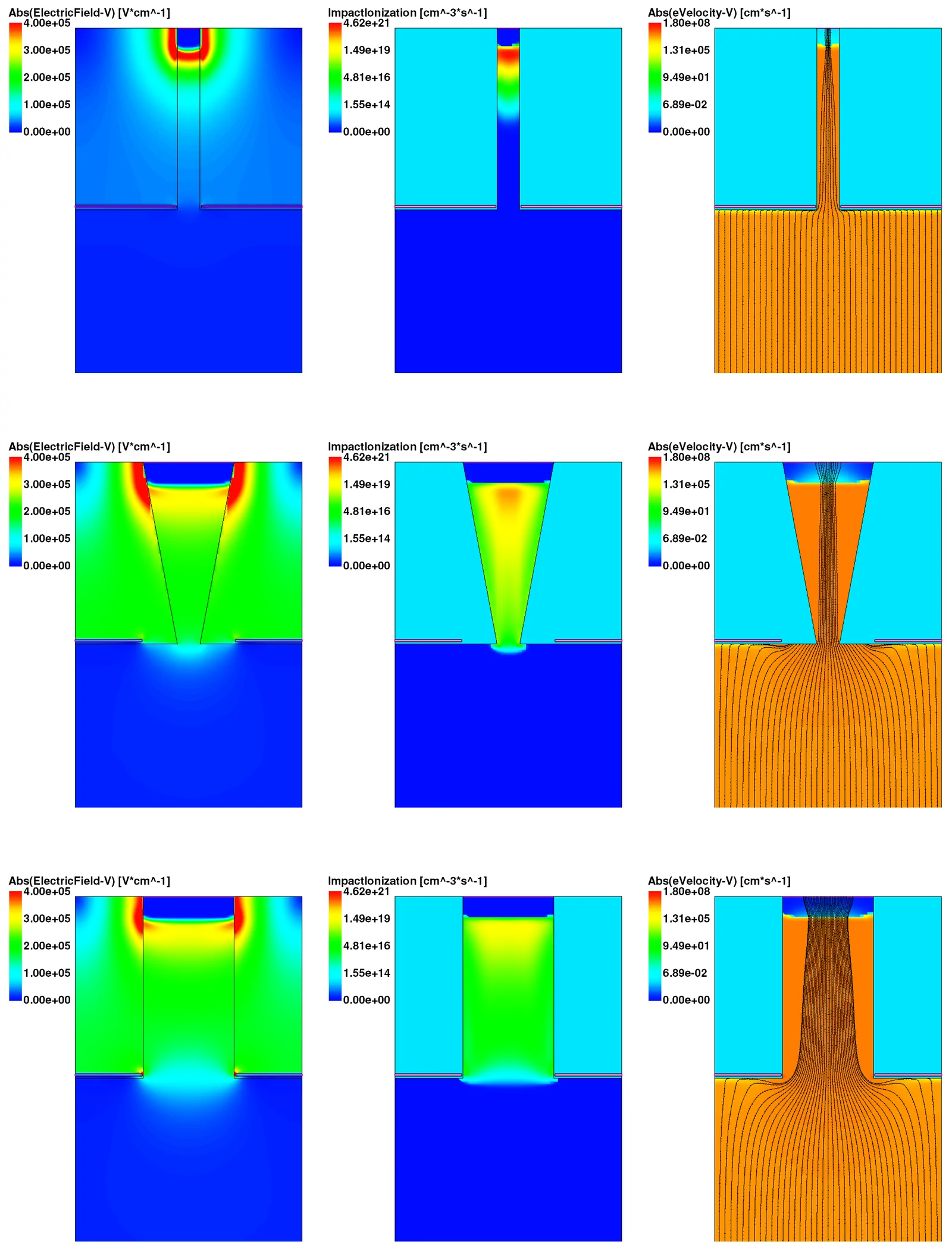
Electric-field, impact-ionisation, and electron-velocity streamline maps for representative single-electrode SiEM geometries. (**Top row**): Baseline small-pillar geometry C 1 μm—R0.05 at 75 V. (**Middle row**): Largest tapered-pillar geometry T 4 μm—R0.05 at 165 V. (**Bottom row**): Largest cylindrical-pillar geometry C 4 μm—R0.05 at 150 V. From left to right, the panels show the electric-field magnitude, impact-ionisation rate, and electron velocity. The comparison illustrates how increasing the pillar size reduces field localisation, extends the multiplication region, and improves carrier steering from peripheral pixel regions towards the pillar.

To interpret the differences observed in the quasistationary gain scans, Figure 5 compares the electric-field distribution, impact-ionisation maps, and electron-velocity streamlines for representative geometries and illustrates how the multiplication region evolves as the pillar size is increased.

In the baseline C 1μm—R0.05 geometry, the electric field is strongly concentrated near the top of the pillar, below the readout electrode. As a consequence, the impact-ionisation rate is highly localised near the pillar tip. This differs from the double-electrode configuration in [7], where the field is established between two buried gain electrodes and peaks deeper within the pillar, away from the readout contact. Here, the field is instead generated between the single buried electrode and the grounded readout electrode, shifting its maximum towards the pillar tip. This difference in field topology explains the simpler, more concentrated pattern observed in Figure 5 compared to the double-electrode case.

Moving to the tapered T 4 μm—R0.05 geometry and to the cylindrical C 4 μm—R0.05 geometry, the field becomes distributed more uniformly along the pillar volume. In both cases, the impact-ionisation region is broader and extends over a larger fraction of the pillar volume, with the cylindrical geometry providing the widest and most uniform multiplication region.

The electron-velocity maps provide additional insight into the charge-transport efficiency. In the small-pillar geometry, electron streamlines starting far from the pixel centre are only weakly focused towards the multiplication region, increasing the probability that carriers generated in peripheral regions do not contribute efficiently to the amplified signal. Enlarging the pillar increases the effective collection cross-section and improves the focusing of electron trajectories towards the high-field region. This behaviour is also supported by the electric-field map, which shows a larger lateral field component extending away from the pillar base and guiding the carriers towards the pillar region.

**Figure 6:**
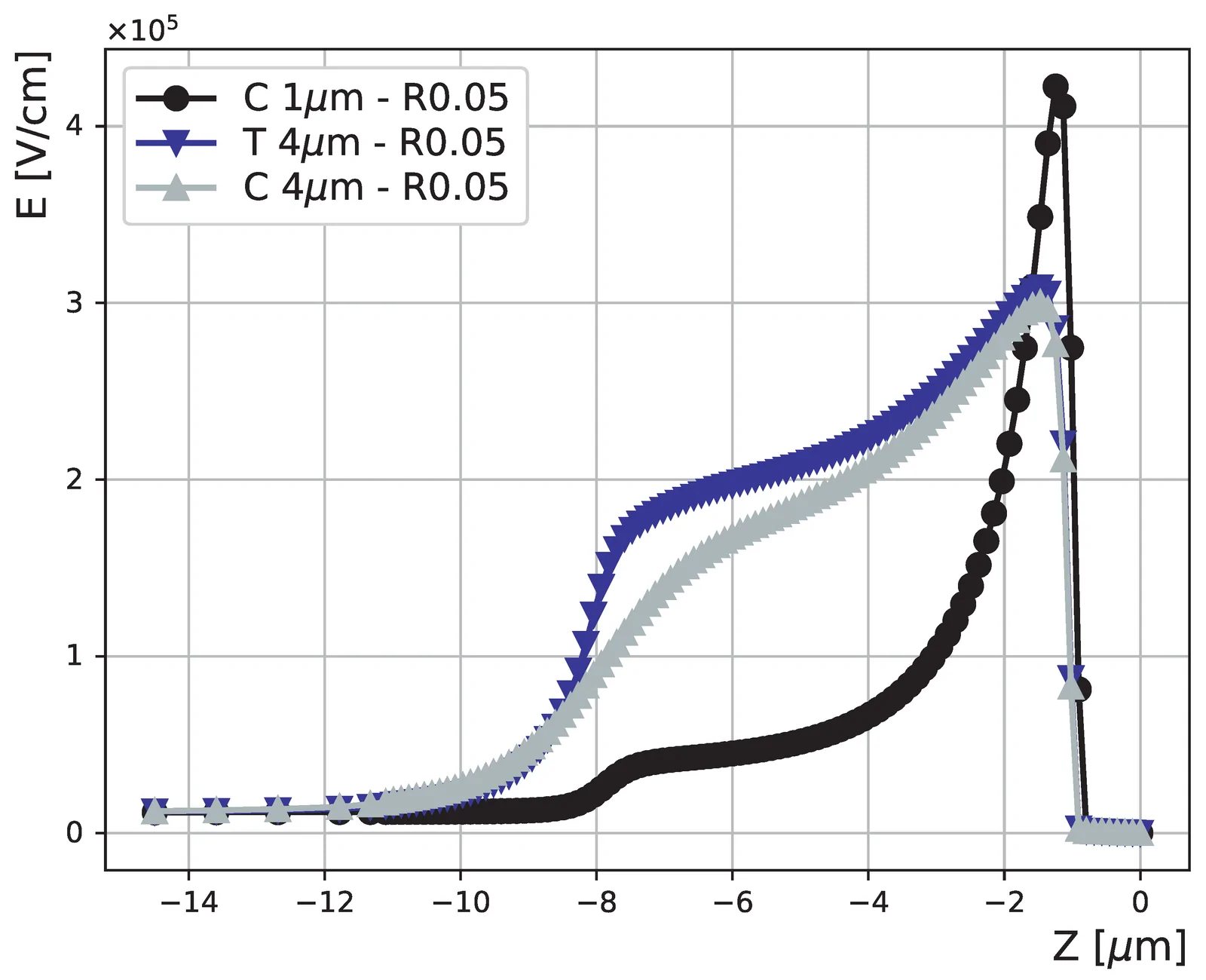
One-dimensional electric-field profile extracted along the pillar axis for the geometries shown in Figure 5. For clarity, only the upper part of the device containing the multiplication region is shown. The comparison highlights the reduction of the peak field and the displacement of the maximum-field region deeper into the pillar for larger-pillar geometries.

Figure 6 further quantifies this effect through a one-dimensional electric-field profile extracted along the central pillar axis. Since the full silicon bulk extends from approximately z=−58μm to z=0μm, only the upper region containing the multiplication region is shown. The profile confirms that enlarged pillars reduce the peak electric field while extending the high-field tail deeper into the pillar, thereby reducing field crowding near the readout side and increasing the high-field volume.

### 4.3. Effective Charge Collection Efficiency Maps

For each geometry, MIP tracks are injected at randomised positions across the pixel cell and the resulting effective charge collection efficiency is evaluated.

**Figure 7:**
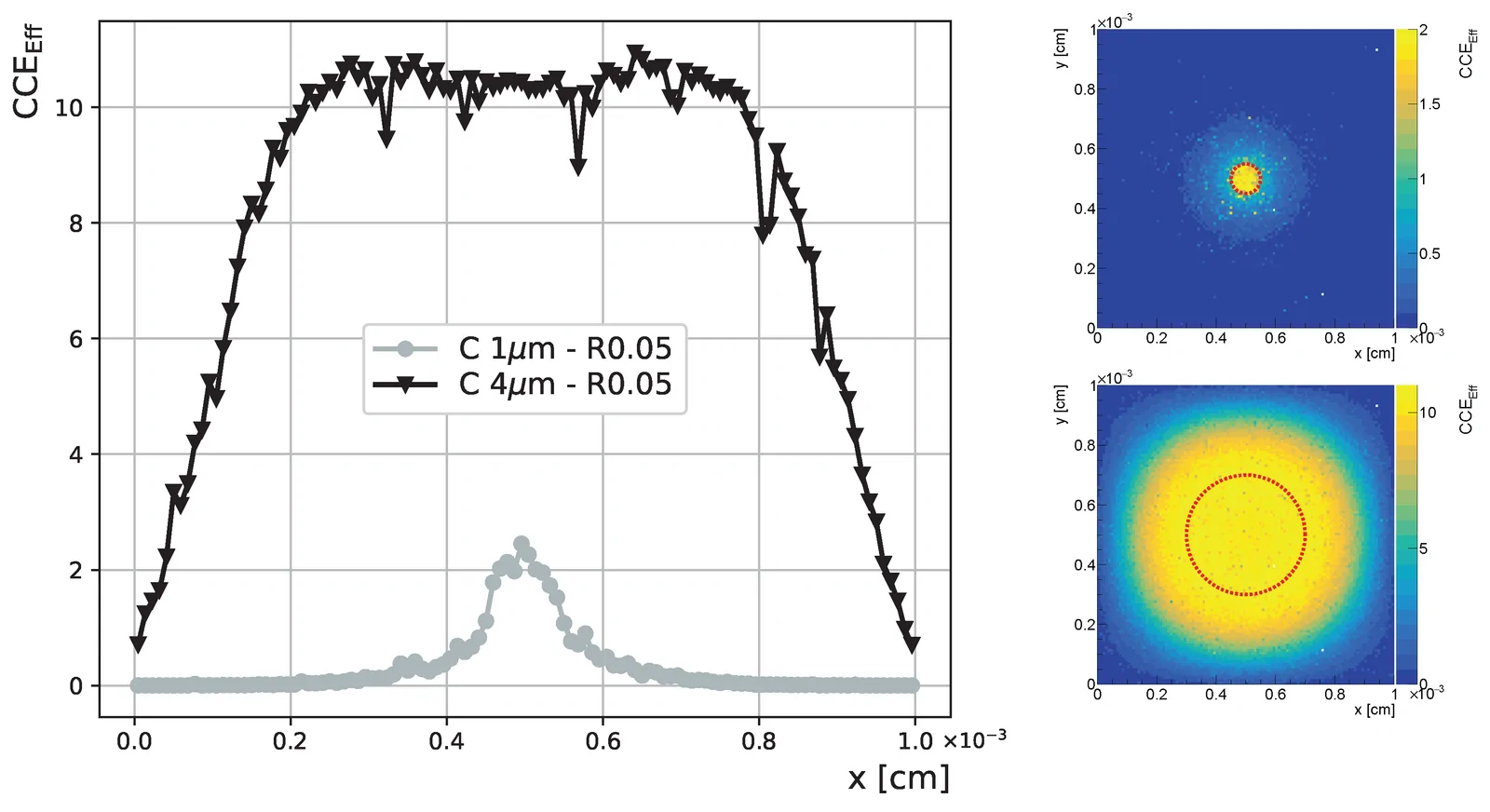
Effective charge collection efficiency for two representative single-electrode SiEM geometries. (**Left**): One-dimensional $CCE_{\mathrm{eff}}$ profile extracted along the pixel centre. (**Right**): $CCE_{\mathrm{eff}}$ maps for the baseline C 1 μm–R0.05 geometry at 75 V (**top**) and for the largest cylindrical C 4 μm–R0.05 geometry at 150 V (**bottom**). The quantity $CCE_{\mathrm{eff}}$ includes both charge-transport efficiency and internal avalanche multiplication. The red circle indicates the pillar ROI used to extract the average pillar value, $\langle CCE_{\mathrm{eff},\mathrm{ROI}}\rangle$.

The resulting $CCE_{\mathrm{eff}}$ maps are shown in Figure 7. The red circle indicates the pillar ROI used to compute the average $\langle CCE_{\mathrm{eff},\mathrm{ROI}}\rangle$ reported in Table 2. Owing to the symmetry of the pixel cell, a direct comparison between the two geometries is also performed by extracting the one-dimensional $CCE_{\mathrm{eff}}$ projection along the central *x* axis. For the baseline C 1 μm—R0.05 geometry, $\langle CCE_{\mathrm{eff},\mathrm{ROI}}\rangle$ is about 2, while the region with $CCE_{\mathrm{eff}}>1$ covers only 1.16% of the pixel area. In contrast, the C 4 μm—R0.05 geometry provides a much broader effective active area. Owing to the larger pillar size and the higher gain voltage reached before breakdown, this geometry achieves $\langle CCE_{\mathrm{eff},\mathrm{ROI}}\rangle =10.4$ and an area efficiency of 84%, producing a substantially larger region with net internal gain.

### 4.4. Time-Centroid Maps and Intrinsic Timing Resolution

**Figure 8:**
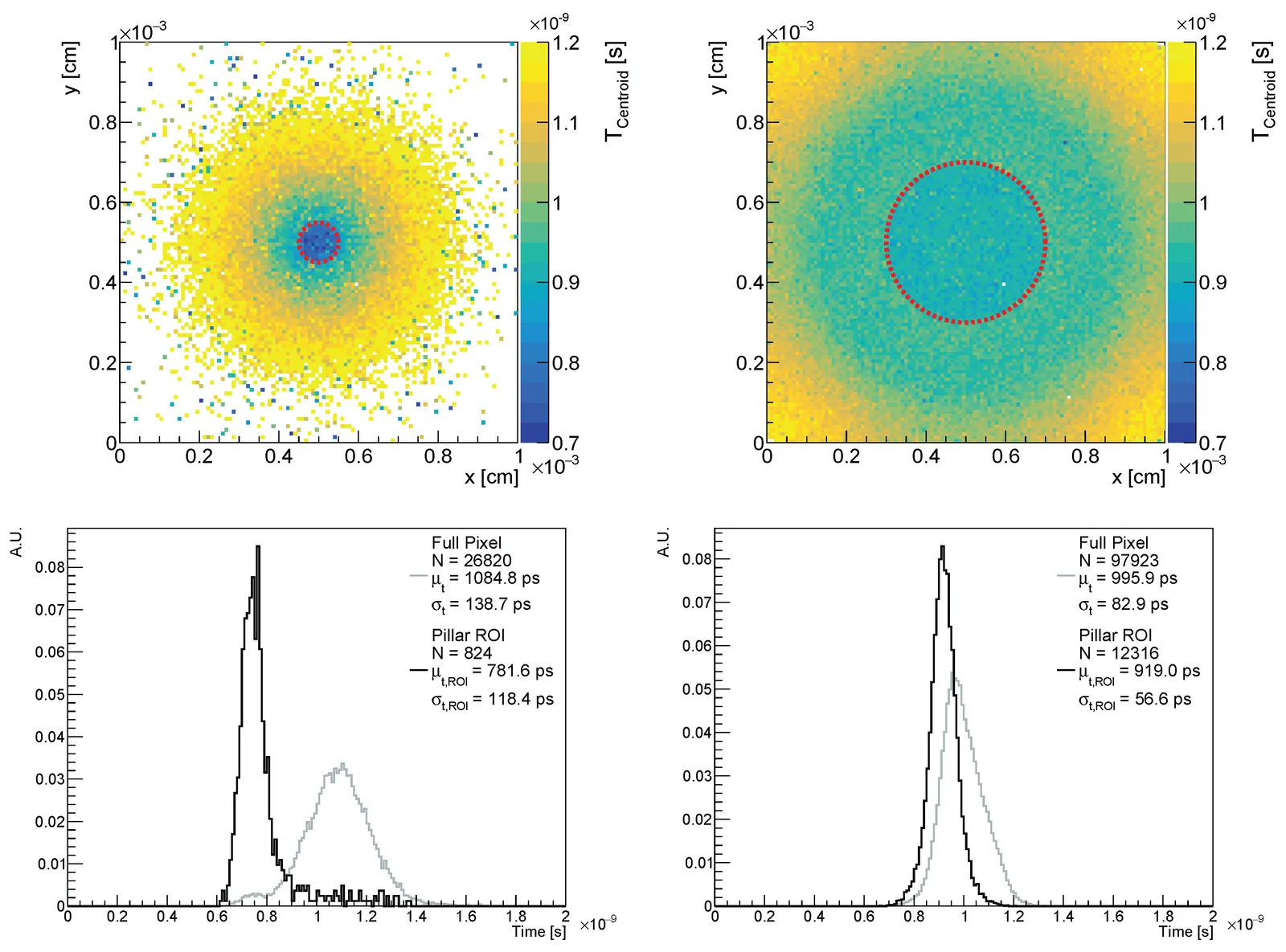
Centroid-time response of two representative single-electrode SiEM geometries. (**Top row**): centroid-time maps over the full pixel area for the baseline C 1 μm—R0.05 geometry at 75 V and for the largest cylindrical C 4 μm—R0.05 geometry at 150 V. (**Bottom row**): corresponding centroid-time distributions extracted over the full pixel area and within the pillar ROI. The distributions are normalised to unit area. The red circle denotes the pillar footprint. All plots are computed for events with collected charge above the 100 $e^{-}$ threshold.

The centroid-time maps for the two representative geometries are shown in the top row of Figure 8. For the C 1 μm—R0.05 geometry, only events generated close to the pillar exceed the collected-charge threshold. Within this active region, the centroid time gradually increases from the pillar centre towards its periphery, indicating longer drift paths and reduced carrier steering efficiency for charges generated farther from the pillar axis. The C 4 μm—R0.05 geometry shows a more uniform centroid-time response, with delayed signals observed mainly near the pixel corners.

The intrinsic timing resolution is extracted as the standard deviation of the centroid-time distribution over the full pixel area, considering only events with collected charge above the 100 $e^{-}$ threshold. The bottom row of Figure 8 shows the centroid-time distributions for the full pixel and for the pillar ROI, normalised to unit area. For the baseline geometry, the intrinsic timing resolution is approximately 138.7 ps. The enlarged geometry provides a more symmetric and uniform timing distribution, with an intrinsic timing resolution of approximately 83 ps. This improvement is a direct consequence of the more uniform charge-collection time across the pixel, which reduces the time skew between the pillar and peripheral regions.

### 4.5. Discussion

The complete set of simulated observables is summarised in Table 2. The results show that the multiplication-region geometry is the primary parameter governing the balance between gain, active area, capacitance, and timing performance.

For the baseline C 1 μm geometries, increasing the gain-electrode retraction leads to an improvement in the timing resolution within the pillar ROI, together with an increase in the readout-to-bulk capacitance. The improvement in the local timing response can be attributed to more effective steering of carriers generated by tracks crossing close to the pillar walls. The capacitance increase is caused by the reduced electrostatic shielding between the readout electrode and the bulk as the gain electrode is retracted. By contrast, the gain onset, area efficiency, and full-pixel timing response show only limited improvement and remain suboptimal, indicating that the local field concentration is determined primarily by the pillar dimensions rather than by the exact radial position of the gain electrode.

Increasing the pillar size shifts avalanche breakdown to higher gain voltages and substantially enlarges the region contributing to charge multiplication. As a result, both $\langle CCE_{\mathrm{eff},\mathrm{ROI}}\rangle$ and the area efficiency increase significantly. In addition, the largest cylindrical geometry, C 4 μm, provides the best full-pixel timing response, because the enlarged multiplication region improves carrier steering from peripheral regions and strongly reduces the centroid-time skew between tracks crossing near the pillar and tracks crossing the pixel periphery.

The comparison between cylindrical and tapered geometries highlights a different behaviour. Although tapered structures provide higher charge multiplication for some configurations (T 4 μm—R0.05), they also exhibit a degradation in area efficiency and timing performance. This suggests that maximising the local electric field alone is insufficient to optimise the overall detector response, as larger fluctuations in transport paths and multiplication locations can offset the benefits of increased gain.

An additional simulation performed for the C 4 μm—R0.05 geometry with a reduced 25 μm thick bulk provides a first indication of the impact of sensor thickness on the timing performance. To preserve a similar electric-field configuration, the bulk bias was scaled proportionally to the sensor thickness, using 25 V instead of 50 V. While the gain and area efficiency remain largely unchanged, a substantial improvement in timing performance is observed. The pillar-ROI timing resolution improves from 56.6 ps to 34.7 ps, while the full-pixel timing resolution improves from 82.9 ps to 56.2 ps. This behaviour is consistent with the shorter carrier drift paths in thinner sensors, which reduce charge-collection-time fluctuations across the pixel.

Overall, the results show that gain, active area, capacitance, and timing cannot be optimised independently. The multiplication-region geometry plays a central role in determining the overall sensor performance and must therefore be treated as a global design parameter rather than as a gain-enhancement feature alone. Among the investigated layouts, enlarged cylindrical pillars provide the most favourable compromise, combining improved charge collection, higher area efficiency, and reduced timing non-uniformities across the pixel.

Future developments should focus on multiplication structures better matched to the pixel footprint, including square or hexagonal pillar geometries, as well as on increasing the pillar-to-pitch ratio through smaller pixel pitches. Additional optimisation may be achieved by extending the design phase space to other geometrical parameters, particularly the bulk thickness and pillar height, which are expected to further influence charge-collection uniformity and timing performance.

## 5. Conclusions

A three-dimensional TCAD and Garfield++ simulation pipeline has been used to investigate single-electrode SiEM geometries and quantify the impact of the multiplication-region design on charge collection and intrinsic timing performance.

The simulations show that the pillar geometry is the dominant parameter controlling the detector response. In the investigated single-electrode geometries, preserving a highly localised multiplication region is not necessary to achieve the best overall detector performance: small pillars produce highly concentrated electric fields, but their response remains confined to a limited fraction of the pixel area. Enlarged cylindrical pillars instead extend the multiplication region, improve carrier steering, and provide higher area efficiency with better full-pixel timing uniformity.

Among the investigated configurations, the C 4 μm—R0.05 geometry provides the most favourable balance, achieving an area efficiency above 80%, an intrinsic full-pixel timing resolution of approximately 83 ps, and a gain above 10. Additional simulations indicate that the timing resolution can be further improved down to 56 ps by reducing the sensor thickness from 50 μm to 25 μm.

These results identify enlarged cylindrical multiplication pillars as a promising direction for future SiEM demonstrators. Future developments should therefore aim to increase the pillar-to-pitch ratio and explore geometries better matched to the pixel footprint, such as square or hexagonal pillars, possibly combined with smaller pixel pitches. Future studies will also investigate thinner bulk substrates for improved timing performance, different pillar heights, radiation damage, and the impact of realistic front-end electronics on detector performance.

## Figures and Tables

**Table 1:** SiEM single-pixel geometry configurations. The compact label indicates the pillar shape (C, cylindrical; T, tapered), the top pillar diameter, and the electrode retraction R, all expressed in μm. For tapered geometries, the bottom pillar diameter is fixed to 1 μm.

Label	Top Diameter [μm]	Bottom Diameter [μm]	Electrode Retraction [μm]	Comment
C 1 μm—R0.05	1	1	0.05	Baseline
C 1 μm—R0.5	1	1	0.5	Retraction
C 1 μm—R1.0	1	1	1.0	Retraction
C 1 μm—R1.5	1	1	1.5	Retraction
T 2 μm—R0.05	2	1	0.05	Tapered
C 2 μm—R0.05	2	2	0.05	Cylindrical
T 3 μm—R0.05	3	1	0.05	Tapered
C 3 μm—R0.05	3	3	0.05	Cylindrical
T 4 μm—R0.05	4	1	0.05	Tapered
C 4 μm—R0.05	4	4	0.05	Cylindrical

**Table 2:** Summary of the main simulated observables for the investigated single-electrode SiEM geometries. The Bulk column denotes the sensor thickness. $C_{\mathrm{bulk},\min}$ is the minimum readout-to-bulk capacitance and $\eta_{\mathrm{area}}$ is the fraction of the pixel area with $CCE_{\mathrm{eff}}\geq 1$. The average $\langle CCE_{\mathrm{eff},\mathrm{ROI}}\rangle$ and $\sigma_{t,\mathrm{ROI}}$ are evaluated within the pillar ROI, while $\sigma_{t}$ is evaluated over the full pixel area for events with collected charge above the 100 $e^{-}$ threshold. The column $V_{\mathrm{gain},\max}$ indicates the highest simulated gain voltage before breakdown and the bias point used for the Garfield++ transient simulations.

Geometry	Bulk [μm]	$V_{\mathrm{gain},\max}$ [V]	$C_{\mathrm{bulk},\min}$ [aF]	$\langle {\mathrm{CCE}}_{\mathrm{eff},\mathrm{ROI}}\rangle$	$\eta_{\mathrm{area}}$ [%]	$\sigma_{t,\mathrm{ROI}}$ [ps]	$\sigma_{t}$ [ps]
C 1 μm—R0.05	50	75	0.016	1.99	1.16	118.51	138.75
C 1 μm—R0.5	50	75	0.068	2.40	1.75	105.29	138.72
C 1 μm—R1.0	50	75	0.180	2.23	1.62	94.84	133.54
C 1 μm—R1.5	50	75	0.340	2.14	1.61	80.48	131.52
T 2 μm—R0.05	50	110	0.120	6.22	13.52	70.22	120.80
C 2 μm—R0.05	50	110	0.180	6.09	19.38	51.63	121.65
T 3 μm—R0.05	50	140	0.420	6.37	19.68	63.61	113.66
C 3 μm—R0.05	50	135	0.710	13.40	59.17	56.49	113.39
T 4 μm—R0.05	50	165	1.400	16.71	38.42	63.42	111.03
C 4 μm—R0.05	50	150	1.850	10.38	84.01	56.59	82.90
C 4 μm—R0.05	25	150	3.694	10.26	79.43	34.70	56.20

## Data Availability

Data is available by contacting corresponding author who will provide access to a shared data folder within a maximum of 10 working days.

## Abbreviations

The following abbreviations are used in this manuscript:

CNM	Centro Nacional de Microelectrónica
DRIE	Deep Reactive Ion Etching
HL-LHC	High-Luminosity Large Hadron Collider
LGAD	Low-Gain Avalanche Detector
MIP	Minimum-Ionising Particle
PAI	Photo Absorption Ionisation
ROI	Region of Interest
SDE	Sentaurus Device Editor
SDEVICE	Sentaurus Device
SiEM	Silicon Electron Multiplier
TCAD	Technology Computer-Aided Design
